# 

**Published:** 1990-12

**Authors:** 


### Books of interest to GPs

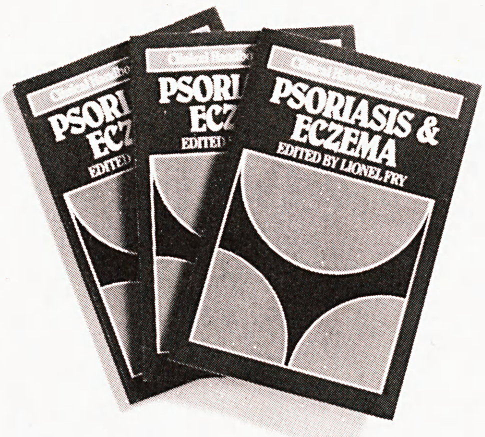

### PSORIASIS AND ECZEMA

Edited by Lionel Fry, BSc, MD, FRCP, MRCS
Consultant Dermatologist, St. Mary's Hospital, London, UK

'For the interested general practitioner or young hospital doctor, it would certainly provide an excellent guide to the clinical features and management of these common skin conditions.' *Australasian Journal of Dermatology*

**Contents:**
Psoriasis: Clinical Features. The Treatment of Psoriasis. The Aetiology and Pathegenesis of Psoriasis. Eczema: Clinical Features. The Management of Eczema. The Aetiology of Aczema. Index.

*210 pages. 216 mm x 138 mm. Papercover. Fully illustrated in colour. ISBN 1 85457 002 1. 1989. £15.00*

### HEART FAILURE

Edited by Gerald Sandler, MD, FRCP, Consultant Physician, District General Hospital, Barnsley, UK and John Fry, CBE, MD, FRCS, FRCGP, General Practitioner, London

**Contents:**
The Pathophysiology of Heart Failure. Clinical Aspects of Heart Failure. Management of Heart Failure. Surgical Management of Heart Disease. Index.

*100 pages. 216 mm x 138 mm. Papercover. ISBN 1 85457 008 0. 1990. £10.00*

### SECOND OPINIONS IN INTERNAL MEDICINE

Gerald Sandler, MD, FRCP, Consultant Physician, District General Hospital, Barnsley, UK

John Fry, CBE, MD, FRCS, FRCGP, General Practitioner, London

Wesley Fabb, General Practitioner, Melbourne, Australia, Secretary of WONCA **and**

Martin Godfrey, MB ChB, MRCGP, Group Medical Editor, Haymarket Publications, General Practitioner, Beckenham, UK

This book is for family medical trainees and their teachers and for teachers of medical students in discussing referrals from family doctors.

**Contents:**
Jaundice. Resistant Hypertension. Breathlessness. Persistent Dyspepsia. 'Mrs Never Well'. Headaches. Swollen Legs. Multiple Joint Pains. Chest Pain. 'Funny Turns'. Dizziness. Back Pain. Index.

*150 pages. Papercover. ISBN 1 85457 019 6. 1990. £12.50*

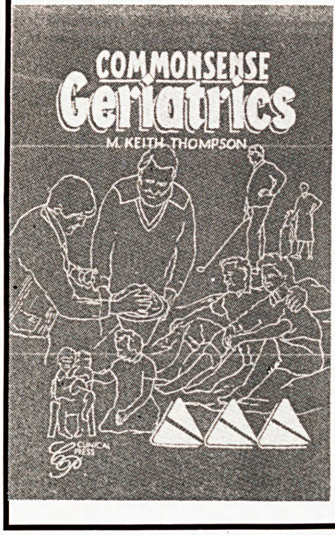

Keith Thompson, MB ChB, FRCGP, DObstRCoG, General Practitioner, London, Examiner for the Diploma in Geriatric Medicine

'Keith Thompson's book should become the keystone of teaching in geriatrics, not only in vocational training for general practice, but also for the growing specialty of hospital and community geriatrics and not only for doctors, but also for nurses, health visitors and social workers.' *Update*

**Contents:**
First Principles. Population and Population Structure. Old People in the Practive. Basic Principles. Attitudes in the Care of the Elderly. Special Difficulties with Older Patients. Practice Organisation. The Team Concept. The Examination. The Investigation. Health Education and Follow up.
The Ageing Process. Common Clinical Problems. Specialty Aspects. The Whole Person. Index

*150 pages. 216 x 138 mm. Hardcover. Fully illustrated. ISBN 1 85457 007 2. £12.50*

*Second edition*

### REGAINING BLADDER CONTROL

Eileen Montgomery, MCSP

This important patient handbook contains a planned series of exercises aimed at restoring control of the bladder. The anatomical causes of incontinence are clearly explained and illustrated. The book also deals with correct breathing and a balanced diet which will not lead to constipation or to being overweight.

*28 pages. Soft cover. 11 line drawings. ISBN : 1 85457 003 X*

*Sold in single copies (£2.50) or in packs of 10 copies. (£20.00)*

**ORDER FORM**

Please order from your usual bookseller or direct from Clinical Press Ltd., c/o Gazelle Book Services, Falcon House, Queen Square, Lancaster, LA1 1RN, UK

Please send me

........... copy(s) of ...................................................................

........... copy(s) of ...................................................................

........... copy(s) of ................................................................... **Prepaid orders or credit-card orders will be supplied post free**

I enclose a cheque for ...................... (Payable to Gazelle Book Services)

OR I authorise you to charge my Access/Barclaycard/American Express credit account

Card: ............................... Number: ............................ Expiry Date: ........................... Signature: ...............................

OR Please invoice me at list price plus £1.50 per copy postage and packing

Name: (Please Print) ...................................................................

Address: (Please Print) ...................................................................

**Editorial Committee**
Chairman of Editorial Board, Professor John Farndon
Editor—Mr M. G. Wilson
Assistant Editors—Dr Paul Goddard, Dr Alison Round
Secretary—Mr Clive Ward
Members—Dr John Burton, Dr Kenneth Southgate

**Editorial Office**—*The Red House, 40 Coombe Lane, W.O.T., Bristol BS9 2BJ*
*Tel 0272 682320*

**Correspondents**
Dr Andrew Adam (Taunton)
Mr Martin Crosfil (Truro)
Dr Jack Davies (Bristol)
Professor Denis Pereira Gray (Exeter)
Dr Jose Jancar (Bristol)
Dr Michael Oliver (Barnstaple)
Dr Michael Whitfield (Bristol)

**Associated Societies**
Bristol Medico-Chirurgical Society
Clinical Society of Bath
Severn Faculty of the Royal College of General Practitioners
South West of England and Wales Society of Dermatology
South West Obstetricians and Gynaecologists Club
South West Orthopoedic Club
South West Paediatric Club
South West Radiologists Club
South West Vascular Surgeons
Surgical Club of South West England
Tamar Faculty of the Royal College of General Practitioners
Wessex Association of Clinical Pathologists
West Country Chest Physicians Club
West Country Physicians Club

Members of the above organisations receive the journal as part of their membership subscription.
Annual Subscription Rate £25.00 including surface postage.

**Published by**
Clinical Press
*15 Bucklands Drive,*
*Nailsea,*
*Bristol BS19 2PH*

**Typesetting by**
*Apek Typesetting*
*Avon House,*
*Nailsea,*
*Bristol BS19 2DJ*

**Printed by**
*Sutton Print, Paignton*

**THE WEST OF ENGLAND MEDICAL JOURNAL acknowledges with gratitude the help it receives from NUFFIELD HOSPITALS**

### WEST OF ENGLAND MEDICAL JOURNAL

**DECEMBER 1990** ISSN: 0960 6440

**Volume 105(iv)**

Copyright © West of England Medical Journal Co Ltd

**Contents**

1883–1990 Vols 1–105

**NOTICE TO CONTRIBUTORS**
Contributions are invited especially from West of England authors on a variety of subjects, e.g. Original articles relating to the practice of medicine, reports on the contemporary medical scene, obituaries, historical accounts, recollections of colleagues and events worthy to be remembered and liable to be forgotten. An article of 2,000 words with 2 illustrations will occupy 2 pages and this is a suggested, though by no means an obligatory length. Articles are preferred in the form proposed by the International Committee of Medical Editors (Vancouver System)—pamphlet obtainable from Editor of BMJ., BMA. House, Tavistock Square, London, WC1H 9JR. Please send two copies retaining a further copy for your reference. **Articles published in this Journal are listed in Index Medicus.**

### INDEX to Volume 105 nos i to iv 1990

Only the first two authors named on each paper are indexed

Adam, A.E., 28, 68
Alderson, D., 98
Allen, D.S., 116
Al-Okati, D., 100
Alzheimer's Disease, MRI in, 74
Ancient Egypt, Mummification in, 119
Anthony, P.P., 36
Armstrong, C.P., 64
Arts Co-ordinator in Psychiatric Hospital, 116
Aziz, T.Z., 39

Baker, A.R., 99
Ball, D.E., 97
Bande, S., 128
Bannister, G.C., 66, 96
Barghouty, N.M. El., 128
Barlow, A.P., 127
Bartlett, R.J., 66
Bartolo, D.C.C., 67, 99, 100
Bateson, M.C., 64
Bathurst, N.C.G., 127
Bayes Theorem for Clinicians, 106
Bell, J., 43
Belsey, R., 102
Berry, P.J., 35
Besterman, T., 126
Bickley, D., 15
Bird, D.R., 130
Bloom, P., 120
Boble, V.R., 129
Boeree, N.R., 66
Bone, Q., 125
Bosley, A.R.J., 33
Braddon, F.E.M., 63
Breast Cancer Screening, 22
Briggs, J.C., 70
Brimblecombe, F., 30
Browning, J., 124
Burton, J.L., 12, 27

Cancer, and Fats, 18
Carcinoma of Pancreas and Selectron Brachytherapy, 20
Carey, M.C., 62
Case, R.D., 96
Cataract Patients, Intra-ocular Lenses for, 42
Cavanagh, P.M., 67
Cave, A.J., 103
Cerebellar Glioblastoma Multiforme, 39
Cervical Cancer, Screening, 77
Chapman, A., 51
Child Health, in South West England, 30
Children, Coronaries and Cholesterol, 7
of the Nineties, 80
Clarke, R.J., 127
Clinical Pathologists Assoc., Wessex Branch,
November 1989 Meeting, 93
December 1988 Meeting, 34
Clyne, C.A.C., 47, 130
Collin, J., 131
Collins, C.D., 67, 128
Colman, V., 84
Composer, West Country, 55
Coronaries, Cholesterol and Children, 7
Cosgrove, C., 130
Cowling, P., 94
Crosfil, M., 91
Currie, I., 95
Curtis, H., 36
Czerniewski, I.W.D., 85

Dalferman, T.G., 126
Dankwa, E.K., 35
Dartmoor, 57
Davies, A., 130
Davies, J.D., 92, 93
De Jong, P., 125
Dent, J.C., 93
Dermatology, and Diet, 12
Dhundee, J., 94
Diet and Dermatology, 12
Doris, J., 94
Dowling, A., 55
Downs's Syndrome, Prevention in SW Region, 15
Dumoulin, J.G., 92
Duthie, G.S., 99, 100

Earnshaw, J.J. 130
Eastwood, D.M., 66, 97
Elderly Population, Explosion in, 84
Eslick, M.A.R., 127
Eyre-Brook, I., 68
Falconer, T., 125
Farndon, J.R., 99
Fats and Cancer, 18
Feneley, R.C.L., 57
Fowler, A., 129
Freeman, R., 124

Gargan, M.F., 96
General Practitioners, Royal College Fellowship, 110
Gepi-Attee, S., 72
Gingell, J.C., 72
Goddard, P., 43
Golby, M., 20
Golding, J., 80
Gotley, D.C., 64, 97
Greene, W.J., 47
Green, T.P., 95, 129
Griffiths, D.A., 126
Griffiths, H.E.D., 91

Habib, N.A., 18
Hall, G.H., 106
Hall, M.S., 24
Hamer, A.J., 99
Hawkins, K., 114
Haworth, J.M., 127
Heaton, K.W., 63
Hepatobiliary Disease, Recent Advances in, 62
HIV, Paediatric Admissions in Zaire, 83
Hip Replacement, To Ski or Not to Ski?, 121
Hobbs, R.J., 95
Horrocks, M., 131
Hosking, S., 116
Humphrey, T., 36

Imaging of Intracranial Tumours, 111
Insulinomas, Intra-operative Ultrasound in, 47
Intra-ocular Lenses for Cataract Patients, 42
Intracranial Tumours, Imaging of, 113

Jancar, J., 25, 89
Jeyasingham, K., 98
Johnson, D.P., 97
Johnson, S.A.N., 68
Johnston, S., 28
Jones, D.A., 96
Jones, L., 95
Jones, S.M., 67
Jung, Carl, Memoir of, 122

Kelly, R.W., 125
Khoo, D.E., 18
Kulkarni, R., 114
Kvriakose, M., 111

Laidlaw, A., 120
Lamont, P., 131
Lithium Toxicity, Management of, 85
Lloyd, George, West Country Composer, 55
Lloyd, J.K., 7
Lumb, G.N., 67

MacGowan, A.P., 35
Magee, T.R., 131
Magnussen, P.A., 66, 95
Majkowski, R.S. 95
Mason, M.C., 131
McConnell, A.A., 34, 93
McCoy, D.R. 94
McDermott, A., 15
McNulty, C.A.M. 93
MDP, Pulmonary Uptake of, 43
Medical Records, A New Approach to, 24
Medicine, Art of, Teaching and Research in, 103
of the People, 51
Mentally Handicapped, Thyroxine, Osteoporosis and Fractures in, 25
Morgan, A.P., 64
MRI in Alzheimer's Disease, 74
Mummification in Ancient Egypt, 117

Nankivell, C., 100
Newington, D.P., 66
Nicoll, J.A.R., 36
Norman, J., 125

O'Boyle, P.J., 68
O'Dwyer, K., 1129
Obituary, Geoffrey Molyneux Fitzgibbon, 87
Gordon Heron, 29
Obstetricians and Gynaecologists Society (SW),
May 1990 Meeting, 124
Operative Surgery, Teaching of, 107
Owens, R.W., 64

Padwick, M., 124
Paediatric HIV Infection in Zaire, 83
Pancreas Carcinoma of, and Selectron Brachytherapy, 20
Park, R., 83
Parry, A., 66
Payne, G.S., 126
Pearse, M.F., 128
Penile Rods, 71
Pereira Gray, D., 24, 110
Placental Abruption and Prostaglandin E2, 114
Plymouth Worthies (I) 3, (II) 48
Population, Elderly, Explosion in, 84
Prostaglandin E2, Placental Abruption following, 117
Pulmonary Uptake of MDP, 43

Radiology, First Bristol Course, November 1989
Ramarakha, P., 130
Ramsahoye, B.H., 94
Referee, Medical, Up and Downs of, 70
Reilly, M., 3, 48
Rigby, H.S., 35, 93
Rodgers, A., 22, 77
Rooker, G.D., 129
Round, A.P., 106
Rowland, C.G., 20
Russell, G.A., 35

Saline Infusion and Amiloride in Lithium Toxicity, 85
Sampath, S., 128
Sarsfield, P., 36
Screening, Breast Cancer, 22
Cervical Cancer, 77
Scriven, M.W., 96
Seal, P.V., 65
Shields, D.A., 131
Short, J.A., 85
Sluglett, J., 117
Smith, E.J., 129
Soap Gets in Your Eyes, 120
South West Obstetricians and Gynaecologists Society,
May 1990 Meeting, 124
S.W. Orthopaedic Club,
Spring 1989 Meeting, 65
Autumn 1989 Meeting, 95
Spring 1990 Meeting, 128
S.W. Paediatric Club,
May 1989 Meeting, 30
S.W. Vascular Surgeons,
February 1990 Meeting, 130
St. Johnston, J.A., 130
Stanley, P.R.W., 131
Stoddart, M., 39
Stoke Park Research Centre, Diamond Jubilee, 89
Surgical Club of the South West,
Spring 1989 Meeting, 67
Autumn 1989 Meeting, 97
Autumn 1990 Meeting, 126

Taylor, R.Z., 96
Teaching, and Research, Medicine Art of, 103
of Operative Surgery, 102
Tennant, W.G., 131
Thomas, O., 128
Thomson, H., 67
Thomson, J.L.G., 74
Thyroxine, Osteoporosis and Fractures in the Mentally Handicapped, 25
Tyfield, L., 35

Ultrasound, Intra-operative, in Surgery of Insulinomas, 47

Vanden Berghe, L., 96, 129
Varty, K., 130
Vascular Surgeons (SW), February 1990 Meeting, 130
Virjee, J., 127

Ward, A.J., 129
Warren, B.F., 35, 93
Weatherley, C., 96, 129
Webb, A. John., 97
Westgate, J., 124
White, S.M., 33
Whitfield, M.J., 26, 90
Wilkins, D., 130
Wills, M., 98
Wilmshurst, C.C., 130
Wilson, M.G., 34, 121
Wilson, N. 15
Wisheart, J.D., 98
Wyatt, M.G., 131

### The Yearbook of General Practice

### THE MEDICAL ANNUAL 1991

NATIONAL LIBRARY OF MEDICINE

NLM 00954105 3

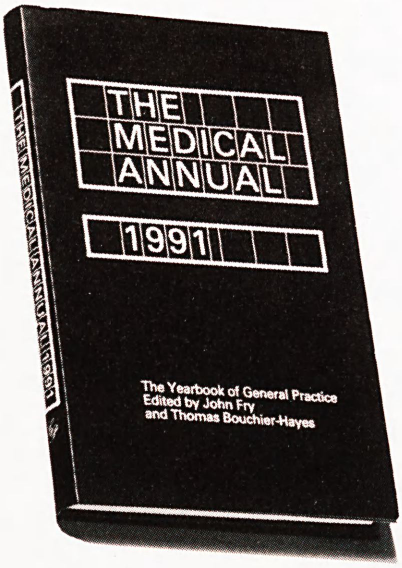

### 107th issue

Edited by: Dr. John Fry, CBE., MD., FRCS., FRCGP, General Practitioner, President of the General Practice Section of the Royal Society of Medicine, Senior Treasurer General Medical Council, Council Member Royal College of General Practitioners

Professor Thomas Bouchier-Hayes, FRCGP., Professor of General Practice, The Royal Army Medical College, Millbank, London, UK

### MEDICAL ANNUAL

**The Year Book of General Practice**

The **Medical Annual** was first published in the 1880's and since that time it has been an established publication among general practitioners and hospital doctors both in the United Kingdom and overseas.

The editors re-introduce this classic publication in an up-to-date form designed to appeal to the new breed of general practitioner. Their objective is to produce an annual review of key developments in medicine that is interesting, lively, useful and attractive as well as being of the highest editorial standards.

**Contents:**
Editorial - The Year in Retrospect GENERAL EVENTS. Medicopolitical Matters. Medicolegal Matters. HEALTH AND DISEASES. AIDS - The Impact on General Practice. Silent Myocardial Ischaemia. Thrombolytic Therapy in Emergency Treatment. Dyspepsia in the Community. Asthma. Diabetes Therapy, New Insulins, Non-insulin Drugs. Laser Hysterectomy. Recent Progress and Advances in Dermatology. Recent Progress and Advances in Viral Hepatitis. Travel Medicine. Depression and Anxiety in General Practice. Sleep Disorders. THERAPEUTICS. he British Pharmaceutical Industry. Recent Advances in Drugs for Cardiovascular Disease. Drugs in Peptic Disease. Drugs for Asthma. Recent Advances in Drugs for Dermatology. Hormone Replacement Therapy. PREVENTION AND HEALTH PROMOTION. Preventive Medicine and Health Promotion. The Practice Nurse Past, Present and Future. Geriatrics. Minor Surgery in General Practice. NEW TECHNOLOGY. Magnetic Resonance Imaging. The Lithotripter. MISCELLANY. Facts - 1990. Personal Musings on General Practice. Great Medical Institutions - The Royal Society of Medicine. Index.

**Editorial Board**

Professor Martin J. Bass (Canada)
Dr. Wesley E. Fabb (Australia)
Professor Pertti Kekki (Finland)
Professor V. Sidel (United States of America)
Professor Philip H. Sive (Israel)
Professor C. Van Weel (Netherlands)
Professor Kerr L. White (United States of America)
Dr. Stephanie A. Amiel
Dr. David Brooks
Mr. J. O. Drife
Professor Sir Michael Drury, OBE
Professor Harold Ellis, CBE
Dr. Lionel Fry
Dr. Eric Gambrill
Dr. Martin Godfrey
Miss Pauline Jeffree
Dr. Gerald Sandler
Dr. Kenneth Scott
Professor George Teeling Smith
Dr. Gregory Wilkinson

**With many eminent contributors**

*220 pages. 30 mm x 150 mm. Casebound. ISSN: 0076 5899 ISBN: 1 85457 021 8. ISSN: 0076 5899. £25.00.*

**ORDER FORM**

Please order from your usual bookseller or direct from Clinical Press Ltd., c/o Gazelle Book Services, Falcon House, Queen Square, Lancaster, LA1 1RN, UK

.......... Please enter my subscription to the **Medical Annual** starting with the 1991 issue and continuing until cancelled.

Please send me copy(s) of the **Medical Annual 1991** at £25.00. **Prepaid orders or credit-card orders will be supplied post free**

.......... I enclose a cheque for ............................ (Payable to Gazelle Book Services)

OR I authorise you to charge my Access/Visa/American Express credit account

Card: .......................................... Number: ........................................ Expiry Date: ........................................ Signature: ..........................................

CLINICAL PRESS

OR Please invoice me at list price [illegible] king Signature: ..........................................

Name: (Please Print) ..........................................

Address: (Please Print) ..........................................

